# Actin Contributes to the Hyperexpression of Baculovirus Polyhedrin (*polh*) and *p10* as a Component of Transcription Initiation Complex (TIC)

**DOI:** 10.3390/v14010153

**Published:** 2022-01-14

**Authors:** Nan Chen, Guanping Chen, Xiangshuo Kong, Xiaofeng Wu

**Affiliations:** 1 College of Animal Sciences, Zhejiang University, Hangzhou 310058, China; chennan9709@163.com (N.C.); 22017026@zju.edu.cn (G.C.); kong_xs@zju.edu.cn (X.K.); 2 Key Laboratory of Silkworm and Bee Resource Utilization and Innovation of Zhejiang Province, Hangzhou 310000, China

**Keywords:** baculovirus, actin, BEVS, hyperexpression

## Abstract

Hyperexpression of *polh* and *p10*, two very late genes, is one of the remarkable characteristics in the baculovirus life cycle. However, the mechanisms underlying the hyperexpression of these two genes are still incompletely understood. In this study, actin was identified as a highly potential binding partner of *polh* and *p10* promoters by conducting DNA pull-down and LC–MS/MS analyses. Inhibiting actin dynamics delayed and decreased the transcription of *polh* and *p10*. Actin interacted with viral RNA polymerase and transcription regulators, and the nuclear import of viral polymerase was inhibited with the disruption of actin dynamics. Simultaneously, the high enrichment of actin in *polh* and *p10* promoters discovered via a chromatin immunoprecipitation (ChIP) assay indicated that actin was a component of the viral polymerase TIC. Moreover, overexpression of actin surprisingly upregulated the expression of luciferase (Luc) under the control of *polh* and *p10* promoters. Taken together, actin participated in the hyperexpression of *polh* and *p10* as a component of TIC. These results facilitate the promotion of the expression efficiency of foreign genes in the baculovirus expression vector system (BEVS).

## 1. Introduction

Baculoviruses are the enveloped, double-stranded (ds) DNA viruses, which primarily infect the lepidopteran insects. They have been widely used as environmentally benign pesticides and recently developed for mammalian gene delivery [1]. One of the amazing phenomena in the baculovirus life cycle is the hyperexpression of two very late genes, *polh* and *p10*, resulting in the production of the occlusion body in which progeny virions are embedded. However, both of these genes are not essential for viral replication and, thereby, can be deleted or replaced by foreign genes. Based on this principle, baculovirus is exploited as the vector for gene expression, and the baculovirus expression vector system (BEVS) is becoming one of the most important expression systems at present [2,3]. Thousands of proteins have been successfully expressed using this system [4], some of which are then developed into vaccines for commercial use [5]. Notably, a major advantage of BEVS is the ease of scale-up from the laboratory to a large-scale production system [6], especially for the use of the *Bombyx mori* nucleopolyhedrovirus (BmNPV)-based expression vector as it can utilize silkworm as the host. However, compared with polyhedrin or *p10*, the expression levels of foreign genes are usually far lower. Hence, it is of great necessity and significance to deeply elucidate the mechanism underlying the hyperexpression of these two very late genes.

Generally, transcription represents the most important initial step in the regulation of gene expression, and transcriptional factors (trans-acting elements) also play important roles. This study aimed to identify proteins binding to *polh* and *p10* promoters of BmNPV, and the results demonstrated that a majority of these proteins were common to both promoters. Among all the identified interacting proteins, actin-4 (Accession: S5M6C1) had the greatest number of unique peptides and was, therefore, utilized for further investigation. Here, we report that actin played an indispensable role in the hyperexpression of *polh* and *p10*.

Previous studies have shown that baculovirus is a prolific manipulator of actin. During the later stages of baculovirus infection, the expression of a subset of baculovirus early gene products induces the nuclear accumulation of monomeric actin (G-actin) and subsequent polymerization for filament (F-actin) formation [7]. Both G-actin and F-actin are in the dynamic state, and actin dynamics must be under tight regulation to guarantee proper functioning [8]. The equilibrium between G-actin and F-actin is precisely controlled by viral gene products [7,9,10,11]. The physical force generated from dynamic actin rearrangements is used for motile cell functions [12]. The host actin polymerization machinery is harnessed by many viral pathogens to assist in effective infection and virus propagation [13,14]. The actin-based gene expression facilitates several stages of baculovirus infection, including ingress of input virus to the nucleus [15], egress of progeny virus from the nucleus, and movement of progeny to the plasma membrane for budded virus (BV) budding [16].

Alternatively, actin plays an integrating role in gene expression. It has been linked to numerous gene expression processes, ranging from gene activation to chromatin remodeling, maintenance of genomic integrity, and intranuclear movement of chromosomes and chromosomal loci. Actin interacts with all the transcribed genes [17], and is copurified with all the three eukaryotic RNA polymerases [18,19,20]. β-Actin, present in the pre-initiation complex, stimulates the transcription by binding to RNA polymerase II [19]. Actin has been extensively reported to have diverse functions in gene transcriptional regulation. Although F-actin appears to function in the processing and packaging of the baculovirus genome [9], little is known about the relationship between actin and viral gene expression. Baculoviruses utilize a combination of host and viral polymerases for viral gene transcription and are the only nuclear replicating DNA viruses. The baculovirus genome is transcribed in four temporally distinct phases, namely, immediate–early, early, late, and very late. The transcription of viral immediate–early and early genes is mediated by host RNA polymerase II [21]. The transition between baculovirus early and late gene transcription depends on DNA replication, which involves an alpha-amanitin-resistant RNA polymerase encoded by baculovirus [22]. The TATA boxes and CAGT motifs promote transcription during the early phases, whereas the ATAAG, GTAAG, and TTAAG motifs promote transcription during the late phase [23]. Late and very late promoters differ primarily in the presence or absence of a burst sequence, a sequence in the downstream transcriptional start site (namely, 90% A + T) [24,25,26,27].

It remains unclear whether actin participates in the transcription of *polh* and *p10* or has a global role in viral gene expression. This study aimed to elucidate the functional roles of actin in the transcription of viral very late genes.

## 2. Materials and Methods

### 2.1. Cells, Viruses, and Plasmids and Antibodies

The BmN cell line was cultured in the SF900 II SFM (Gibco, Waltham, MA, USA) supplemented with 3% fetal bovine serum (FBS, Gibco, Waltham, MA, USA) at 27 °C. The T3 strain of BmNPV was termed as the wild-type (WT) virus. The expression plasmid pIZ-actin-Flag was generated from the pIZ/V5-His plasmid. Actin was amplified from cDNA (the cDNA was reverse transcribed from RNA derived from BmN cells) by using primers actin-F (GGATCCATGTGCGACGAAGAAGTTGCC; the BamHI site is indicated by underline) and actin-R (GAATTCTTACTTATCGTCGTCATCCTTGTAATCGAAGCACTTCCTGTGTAC; the EcoRI site is indicated by underline) and cloned into pIZ/V5-His plasmid using BamHI and EcoRI, generating pIZ-actin-Flag. Rabbit-HA (30702ES20; Yeasen, Shanghai, China) and Mouse-Flag (30503ES20; Yeasen, Shanghai, China) were used in the Co-IP assay.

### 2.2. Pull-Down and Liquid Chromatography–Tandem Mass Spectrometry (LC–MS/MS) Analyses

The probes were amplified from *polh* and *p10* promoters of BmNPV by RCR using biotin labeled primers (Supplementary Table S1). Biotin-labeled *polh* fragment (192 nt, −171~+21) and *p10* fragment (169 nt, −136~+25) were incubated with 100 g streptavidin-coated Dynabeads (Thermo Fisher Scientific, Waltham, MA, USA) in 400 µL binding buffer (10 mM Tris, pH 7.5, 1 mM EDTA, 1 M NaCl, and 0.003% NP40) for 30 min at room temperature under constant and slow rotation. A protein pull-down assay was conducted as described in a previous study [28]. BmN cells were infected with BmNPV at a multiplicity of infection (M.O.I.) of 10. Nuclear proteins from cells infected with BmNPV or uninfected cells were extracted with NE-PER Nuclear and Cytoplasmic Extraction Reagents (Thermo Fisher Scientific). The protein concentration was examined using a BCA Protein Assay Reagent Kit (Thermo Scientific, MA, USA). The unique protein bands were selected for protein identification using LC–MS/MS by Shanghai Applied Protein Technology.

### 2.3. Drug Treatments

BmN cells (1 × 10^6^) were infected with BmNPV at an M.O.I of 10. At 15 h post infection (h p.i.), jasplakinolide (MCE, Monmouth Junction, NJ, USA; 1 µM) and cytochalasin D (EMD Bioscience, Burlington, MA, USA; 1 µg/mL) were added, while the same amounts of DMSO were added in the control group.

### 2.4. Quantitative Reverse Transcription PCR (RT-qPCR)

BmN cells were harvested at the corresponding time points and the total RNA was extracted using the TRIzol™ reagent ((TaKaRa, Dalian, China). Thereafter, the first-strand cDNAs was synthesized using 5 μg total RNA with the TransScript One-Step gDNA Removal and cDNA Synthesis SuperMix (TransGen, Beijing, China). The primers are listed in Supplementary Table S2. qRT-PCR was performed using Hieff^®^ qPCR SYBR Green Master Mix (Yeasen, Shanghai, China). 95 °C for 5 min, then 40 cycles at 95 °C for 5 s, and 60 °C for 30 s were included in the reaction programs. Each sample was triplicated. The 2^−^^ΔΔCt^ method was applied to calculate the relative expression levels of selected genes.

### 2.5. Expression of FLAG-Tagged and HA-Tagged Proteins

Multiple primer pairs are listed in Supplementary Table S3 to construct Flag- and hemagglutinin (HA)-tagged expression bacmids. A HA Tag and a Flag Tag within the primers at C terminus were designed for Flag-tagged and HA-tagged protein expression. The ORFs of viral proteins were amplified from the BmNPV Bacmid and cloned into the pEASY-T1 Simple vector (TransGen, Beijing, China) for sequencing. Thereafter, then ORFs were subcloned into pFastBacHTB vector (Invitrogen Life Technologies, Carlsbad, CA, USA) for the construction of recombinant bacmids according to the manufacturer’s protocol (Invitrogen Life Technologies). Tn7 cassettes from the constructed pFastBacHTB were transferred into the BmNPV bacmid to construct the recombinant viruses Bm-P47-HA, Bm-LEF4-HA, Bm-PK1-HA, Bm-LEF9-HA, Bm-IE1-HA, Bm-VLF1-HA, Bm-VLF-HA, and Bm-actin-Flag. BmN cells were transfected with 2 μg of Bm-P47-HA, or of Bm-LEF4-HA, or of Bm-LEF9-HA, or of Bm-PK1-HA, or of Bm-IE1-HA, or of Bm-VLF-HA, or of Bm-actin-Flag, using 8 μL lipoInsect (Beyotime, Shanghai, China). The cells were replenished with 2 mL of fresh cell culture medium after incubation for 5 h. Thereafter, the cells were incubated at 27 °C, and BV supernatants were collected at 96 h p.i.

### 2.6. Immunofluorescence

Immunofluorescence was performed as described [29,30]. In short, the infected BmN cells with the respective viruses were co-infected on coverslips. The preliminary step was fixation in 4% paraformaldehyde at the indicated time points, subsequently permeabilized in 0.1% TritonX-100. Cells were incubated with rabbit polyclonal anti-Flag and mouse polyclonal anti-HA antibody (dilution 1:500) overnight at 4 °C after blocking in 0.5% BSA/PBS for 1 h. The next step was that cells were incubated with secondary antibodies conjugated with fluorescent labels at a dilution of 1:500 (Alexa 546–conjugated goat anti-rabbit antibody and Alexa 488–conjugated goat anti-mouse antibody (Invitrogen, Carlsbad, CA, USA). The cell nucleus was stained in 4′,6′-diamidino-2-phenylindole (DAPI) (Beyotime, Shanghai, China) for 15 min and examined with a ZEISS LSM 780 confocal laser scanning microscopy.

### 2.7. Co-IP Assay

The Co-IP assay was performed as described in a previous study [31]. Respective proteins were overexpressed in BmN cells. Then, 1 mL cell lysis buffer was added for Western blot and IP. The input was 40 μL of supernatant, which was then incubated with beads coupled with anti-FLAG antibody or anti-HA antibody (Bimake, Houston, TX, USA), while gently blowing and mixing at 4 °C for 24 h. After incubation, the beads were collected and washed with PBS five times. Thereafter, the samples were incubated with 5× loading buffer at 100 °C for 10 min and subjected to SDS-polyacrylamide gel electrophoresis (PAGE) for Western blot examination.

### 2.8. ChIP-Seq and ChIP-qPCR

ChIP assays were performed as previously described, with few modifications [32,33]. ChIP-seq libraries were prepared and sequenced by Wuhan IGENEBOOK Biotechnology Co. Ltd. Given that *polh* and *p10* transcripts are abundant at 48 h p.i., we prepared biologically duplicate ChIP-seq libraries of actin from cells infected with vBm-actin-Flag. Chromatin was harvested, and immunoprecipitations were performed using Flag Tag antibody. In addition, no-Flag immunoprecipitation was used as a negative control. Briefly, 10^7^ BmN cells infected with recombinant virus vBm-actin-Flag 48 h p.i. were collected and cross-linked with 1% formaldehyde for 10 min at 25 °C and then quenched by the addition of glycine to a final concentration of 125 mM. Afterward, samples were lysed on ice to obtain chromatins. Chromatins were sonicated to get soluble sheared chromatin (average DNA length of 200 to 500 base pairs (bp)). One part of the soluble chromatin was saved at −20 °C for input DNA, and the remainder was used for immunoprecipitation by Flag antibody.

Trimmomatic (version 0.38) was used to filter out low-quality reads. Clean reads were mapped to the reference *B. mori* genome using BWA. The same files were converted into bam format using SAMtools. For data scaling, ChIPseqSpikeInFree was used to normalize the ChIP-seq according to the spike-in control. MACS2 was adopted for peak calling using the default parameters. The ChIP-seq data generated in this study has been submitted to the NCBI BioProject database (https://www.ncbi.nlm.nih.gov/bioproject/, accessed on 1 December 2021) under accession number PRJNA735791. In addition, the public ChIP-seq data generated from BmN cells were analyzed by the same method except for the spike process. The immunoprecipitated genomic DNA fragments were amplified using quantitative real-time PCR (qRT-PCR) with primers (Supplementary Table S4). qPCR was performed using Hieff^®^ qPCR SYBR Green Master Mix (Yeasen, Shanghai, China). For the data on each gene, triplicate biological samples and three technical replicates were used.

### 2.9. RNA-Seq

Cell transfection and co-transfection were conducted when the cell density reached 80%. BmN cells were transfected with pIZ-actin-Flag and pIZ/V5-His (as control), then were infected with BmNPV at 12 h post transfection (h p.t.) Total RNA from cells transfected with pIZ/V5-His and pIZ-actin-Flag was isolated and purified using TRIzol™ reagent at 48 h p.i. (TaKaRa, Dalian, China). RNA-seq libraries were prepared as described previously [34] with some modifications. Briefly, the cells were transfected with recombinant vectors pIZ-actin-Flag and pIZ/V5-His with Lipo8000 (Beyotime, Shanghai, China) and then infected with BmNPV at an M.O.I. of 10. Total RNA was extracted at 48 h p.i. using the TRIzol™ reagent (Invitrogen). Approximately 3 μg of RNA per sample was used as the input material for library preparation. NEBNext Ultra™ RNA Library Prep Kit for Illumina (NEB, Ipswich, MA, USA) was used for the construction of libraries, and the index codes were added to attribute the sequences to each sample. Then, the obtained libraries were sequenced on the HiSeq X Ten illumina platform with a paired-end read of 150 bp (LC-Bio Technology CO., Ltd., Hangzhou, China). The raw RNA-seq data quality was evaluated by the FastQC program, and the sequencing adaptors were trimmed by Trimmomatic and only the uniquely mapped reads were preserved, while the BmNPV reads were removed. We aligned reads of all samples to the <research species> reference genome using HISAT2 (https://daehwankimlab.github.io/hisat2/, accessed on 1 December 2021, version: hisat 2-2.0.4) package, which initially removes a portion of the reads based on quality information accompanying each read and then maps the reads to the reference genome. Genes that had two-fold changes (pIZ-actin-Flag group) between the control transcripts (pIZ/V5-His group) and whose p.adjust was less than 0.05 were identified as DEGs. Gene ontology (GO) and Kyoto Encyclopedia of Genes and Genomes (KEGG) pathway were performed to identify the biological pathways altered in response to pIZ-actin-Flag transfection. The RNA-seq data have been submitted to the NCBI BioProject database (https://www.ncbi.nlm.nih.gov/bioproject/, accessed on 1 December 2021) under accession number PRJNA735599.

### 2.10. Transfection and Measurement of Luciferase Activity in Cell Culture

The analysis of the polh and *p10* promoter was conducted using the firefly luciferase reporter. Luciferase was amplified from PGL4.11 by using primers Luc-F-1 (GGATCCGGCAATCCGGTACTGTTGGTAAAGC; the BamHI site is indicated by underline) and Luc-R-1 (GAATTCACGGCGATCTTGCCGCCCTTCTTGGCCTTAAT; the EcoRI site is indicated by underline) and cloned into pFastBacHTB using BamHI and EcoRI, generating pFast-polh-Luc. Luciferase was amplified from PGL4.11 by using primers Luc-F-2 (CTCGAGGGCAATCCGGTACTGTTGGTAAAGC; the XhoII site is indicated by underline) and Luc-R-2 (GGTACCACGGCGATCTTGCCGCCCTTCTTGGCCTTAAT; the KpnI site is indicated by underline) and cloned into pFastBacDual using XhoII and KpnI, generating pFast-p10-Luc. Construction of vBm-polh-luc and vBm-p10-luc was accomplished by transfer of the Tn7 cassette from the constructed pFast-polh-Luc or pFast-p10-Luc into the BmNPV bacmid. The primers are listed in Supplementary Table S5. Cells were transfected with pIZ and pIZ-actin-Flag. After an additional 12 h, they were transfected with a mixture of 20 µg pIZ or pIZ-actin-Flag, then infected with vBm-polh-luc and vBm-p10-luc at an M.O.I. of 10 at 12 h p.t. At 48 h p.i., the cells infected with virus were lysed in 1× passive lysis buffer (Promega, Madison, WI, USA). The luciferase reporter activity was measured using a luciferase reporter assay (Promega) according to the manufacturer’s protocol. The transfections were independently repeated three times. The firefly luciferase activities were normalized against the liquid without color-producing liquid.

### 2.11. Statistical Analyses

Statistical differences were analyzed by Student’s *t* test of GraphPad Prism 7.0 software(Harvey Motulsky, Los Angeles, CA, USA). When *p* value was ≥0.05, differences were considered not significant (ns). When *p* value was <0.05, differences were considered significant. When *p* value was <0.01, differences were considered highly significant.

## 3. Results

### 3.1. Screening of the polh and p10 Promoter-Binding Proteins by DNA Pull-Down Assay and Mass Spectrometry

**Figure 1:**
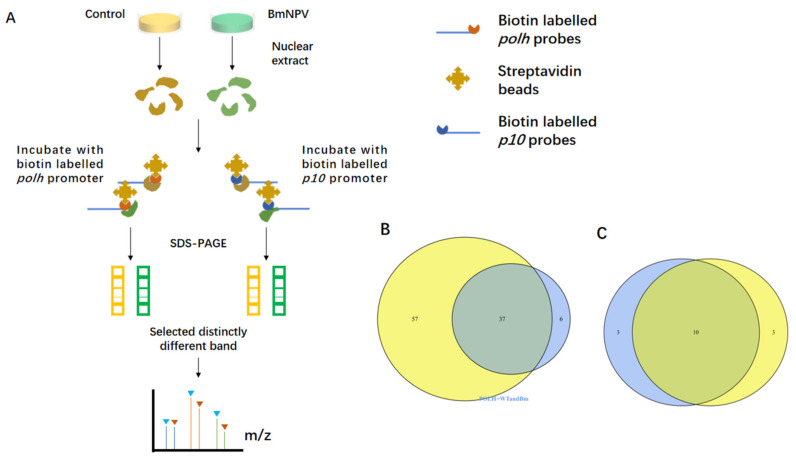
MS analysis of the binding proteins to the *polh* and *p10* promoter. (**A**) Schematic of our approach. (**B**) Venn diagram displaying the summary statistics of the host proteins bound to *polh* promoter and *p10* promoter. Total proteins identified in *polh* are indicated by a blue circle and proteins identified in *p10* are indicated by a yellow circle. (**C**) As in (**B**), summary statistics of the viral proteins bound to *polh* promoter and *p10* promoter.

To identify a comprehensive set of *polh* and *p10* promoter-interacting proteins, this study performed a DNA-protein pull-down assay. As shown in the workflow (Figure 1A), *polh* and *p10* promoters were labeled with biotin and incubated with streptavidin-coated Dynabeads. Later, nuclear extracts from BmNPV-infected and uninfected cells (controls) were extracted, respectively, and incubated with biotinylated *polh* and *p10* promoters to detect the promoter-binding proteins induced by virus infection. Three independent biological replicates were carried out, then distinct the bands in the BmNPV-infected group were selected and identified by on-bead trypsin digestion followed by LC-MS/MS.

Subsequently, *polh* and *p10* were compared, which revealed a general trend that the more abundant proteins (the higher number of unique peptides identified) were common binding partners for each promoter. All these proteins were divided into two categories, including 10 viral proteins and 37 host proteins (Figure 1B,C). A great majority of the identified viral proteins were known as DNA-binding proteins (Supplementary Table S6). For instance, P47, LEF8, and LEF9 were the subunits of viral RNA polymerases for *polh* and *p10* transcription [35]. VLF-1 activated the transcription of *polh* and *p10* by specifically binding to the promoter burst sequence in vitro [36]. To some extent, the above results indicated that the bands we selected were reliable. Intriguingly, numerous novel host proteins were discovered, including several isoforms of actin and ribosome biogenesis regulatory protein. The majority of host proteins were related to RNA processing and gene transcription (Supplementary Table S7).

Actin possesses highly unique peptides and is reported to be involved in many gene expression regulatory processes. Therefore, this study focused on actin for further experiments. Investigating the complex network between actin and the influencing factors of viral transcription may help to illuminate the roles of actin in the hyperexpression of *polh* and *p10*, which will be beneficial for uncovering the underlying molecular mechanisms.

### 3.2. The Actin Dynamics Was Essential for the Transcription of polh and p10

**Figure 2:**
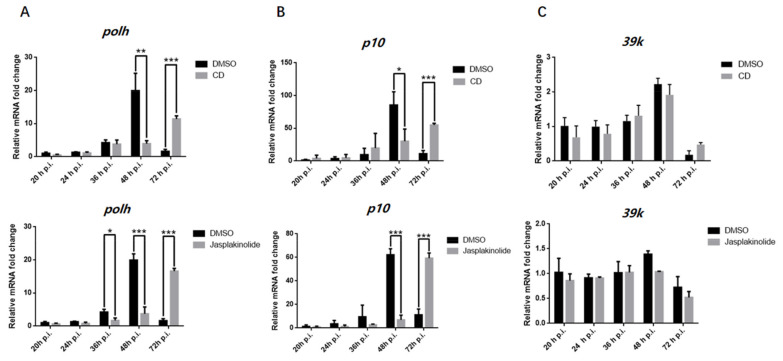
RT-qPCR analysis of the effect on *polh* and *p10* transcription caused by CD and Jas. (**A**) Quantitative real-time PCR analysis of *polh* in BmN cells treated with CD, Jas, or DMSO (Ctrl) (*n* = 3; *, *p* < 0.05; **, p < 0.01; ***, p < 0.001). (**B**) Quantitative real-time PCR analysis of *p10* in BmN cells treated with CD, Jas, or DMSO (Ctrl) (*n* = 3; *, *p* < 0.05; ***, *p* < 0.001). (**C**) Quantitative real-time PCR analysis of 39k in BmN cells treated with CD, Jas, or DMSO (Ctrl) (*n* = 3). The white columns are for the dimethyl sulfoxide (DMSO) treatment as control, the black columns are for CD and Jas treatment. The data indicate the means plus standard errors from three independent assays.

To determine whether actin affected the gene expression of *polh* and *p10*, cells were infected with BmNPV and treated with drugs that prevented G-actin assembly (cytochalasin D, CD) or stabilized F-actin (jasplakinolide, Jas). First of all, we established the timing of nuclear recruitment and polymerization of actin, and found that F-actin began to appear in nuclei at 16 h post-infection (p.i.) (Supplementary Figure S1), which was consistent with a previous study reporting that actin began to accumulate in nuclei at 10–20 h p.i., while G-actin began to polymerize at 2.0 ± 0.4 h after nuclear import [37]. The drugs were added at 15 h p.i. before actin polymerization, then RNA was extracted at the corresponding time point, and RT-qPCR was performed. The mRNA levels of *polh* and *p10* were significantly downregulated at 36 or 48 h p.i. (Figure 2A,B). However, their mRNA levels were increased at 72 h p.i., demonstrating that the transcription of *polh* and *p10* was delayed upon CD and Jas treatment. Conversely, 39k gene transcription was not significantly attenuated with CD or Jas treatment (Figure 2C). These results were consistent with a previous study [38]. Additionally, cells with CD treatment inhibited the amplification of polyhedrin synthesis by at least 8 h [39].

### 3.3. Actin Was Associated and Colocalized with Viral Polymerase

**Figure 3:**
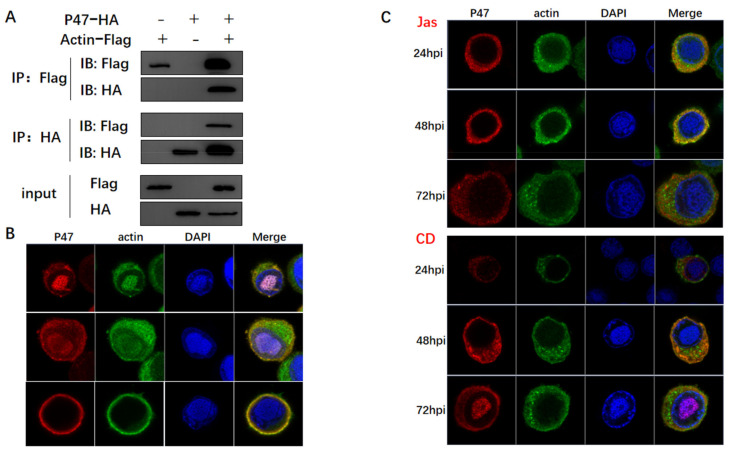

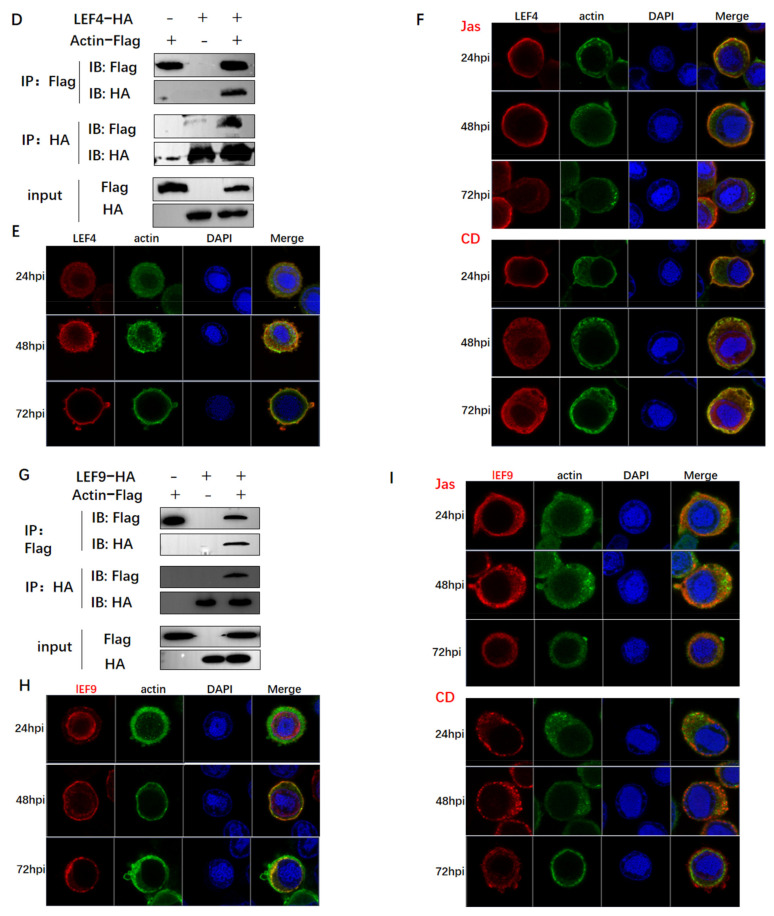
The analysis of the relationship between actin and viral polymerase. (**A**,**D**,**G**) Co-IP assays used to confirm interactions of actin with P47, LEF4, and LEF9. BmN cells coinfected with recombinant viruses vBm-actin-Flag and vBm-P47-HA, vBm-LEF4-HA, and vBm-LEF9-HA were lysed at 72 h p.i. and the proteins were immunoprecipitated with anti-Flag or anti-HA monoclonal antibody. The precipitates (Co-IP) were detected by Western blots with anti-Flag and anti-HA monoclonal antibody. The cell lysates (lysate input) were also examined by Western blots with an anti-HA rabbit polyclonal antibody or an anti-Flag rabbit monoclonal antibody. (**B**,**E**,**H**) Co-localization analysis of actin with P47, LEF4, and LEF9 by confocal microscopy. At the designated time points, BmN cells coinfected with recombinant viruses vBm-actin-Flag and vBm-P47-HA, vBm-LEF4-HA, and vBm-LEF9-HA were fixed, permeabilized, blocked, and incubated with rabbit anti-Flag antibody and mouse anti-HA antibody, followed by treatment with Alexa Fluor 546–conjugated goat anti-rabbit IgG and Alexa Fluor 488–conjugated goat anti-mouse IgG. (**C**,**F**,**I**) As in (**B**,**E**,**H**), at 15 h p.i., cells were treated with CD or Jas and then subjected to confocal microscopy.

Considering that the transcription of RNA polymerase II is dependent on actin, a series of Co-IP assays were conducted to examine the potential association between actin and viral polymerase. In brief, BmN cells were co-infected with two types of viruses, including one expressing Flag-tagged actin and the other one expressing HA-tagged subunits of viral polymerase. BmN cells infected with only one virus were used to confirm the tag protein was expressed, which represented the input group. The four viral subunits of LEF-4, LEF-8, LEF-9, and p47 were found to encode components of viral RNA polymerase [35]. At 72 h p.i., cells were lysed, and proteins were immunoprecipitated with an anti-HA mononuclear antibody. The expression of both tagged proteins was confirmed by WB using the respective antibodies. According to the results, actin interacted with three subunits (Figure 3A,D,G). Confocal microscopy was conducted to further analyze the interactions, which determined the cellular colocalization of actin with viral polymerase. The three subunits of viral polymerase showed a common distribution, which were localized in the nucleus and cytoplasm of infected cells at 24 h p.i., and mostly in the cytoplasm and nuclear membrane until 72 h p.i. (Figure 3B,E,H). This subcellular localization was correlated with the function of viral RNA polymerase in initiating late gene transcription in the nucleus. Actin and viral polymerase displayed a dynamic distribution from the nucleus to the cytoplasm, the colocalization signals were variably concentrated in the nucleus and cytoplasm.

The infected cells were treated with CD and Jas at 15 h p.i. and subjected to confocal microscopy at corresponding time points. At 20 h p.i., three subunits of viral polymerase were found to be translocated to the cytoplasm, and nuclear colocalization was attenuated (Figure 3C,F,I), deviating from its usual nuclear localization in normal cells. This happened accompanied by altered actin localization, with actin concentration in cytoplasm and disruption of actin dynamics. At 72 h p.i., it was not completely absent in the nucleus, probably due to the slight drug effect or the presence of other factors triggering its movement. This also explained why drug treatment delayed gene expression rather than completely suppressing protein expression. The above results demonstrated that actin and viral polymerases interacted in the nucleus early after infection, and actin dynamics was indispensable for nuclear import and recruitment of viral polymerase. Therefore, we deduced that actin dynamics induced nuclear translocation of the viral polymerase, which powered the recruitment of the viral polymerase to the promoter. Our results were in agreement with the idea that nuclear actin dynamics regulated the localization of RNA polymerase II [40].

### 3.4. Actin Was a Component of TIC for polh and p10 Transcription

**Figure 4:**
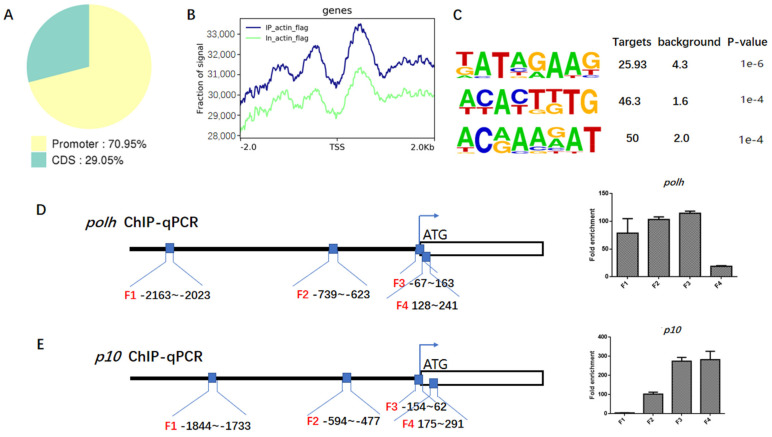
ChIP-seq and ChIP-qPCR analysis. (**A**) Statistics of distribution of actin-binding sites in the virus genome. (**B**) Distribution of actin-binding sites in the genic regions of the recombinant virus Bm-actin-Flag. (**C**) The top four most highly enriched motifs of actin in viral gene. Information regarding the most significant motifs of viral genes identified in the actin-binding peaks with Multiple EM for Motif Elicitation (MEME). (**D**,**E**) Verification of the ChIP-Seq results by ChIP-qPCR including four regions upstream of *polh* and *p10* TSS. The primer pairs used in the RT-qPCR assay are represented with black lines (black boxes from left to right are F1 to F4). Enrichment of target DNA was represented as a percentage of input DNA. The values in each column are the means of three independent replicates and error bars represent the SEM.

To further elucidate the relationship between actin and the viral gene, ChIP-seq analysis was conducted and 54 actin-binding sites were identified in viral genome (Supplementary Table S8). As revealed by genomic annotation of ChIP reads, 70.95% of actin sites resided in the promoter regions of annotated genes (Figure 4A,B), suggesting that actin might be involved in an early step of viral gene transcription. The transcription of viral late and very late genes started in the conserved late and very late gene promoter motif sequence (A/T/G) TAAG. The TAAG sequence is an essential element for both *polh* and *p10* promoters, which is also a common binding motif for viral polymerase [24]. The strength of hyperexpression of very late genes is dependent on an A/T rich sequence present in the downstream sequences of very late promoters [26]. As identified by de novo motif analysis, the most significantly enriched motif at the actin sites contained a major consensus sequence ‘TATAGAAG’ (Figure 4C). To some extent, this verified that actin participated in the transcription of viral polymerases.

ChIP-seq results were further confirmed by ChIP-qPCR analysis, which showed that actin was markedly enriched into the *polh* and *p10* promoter regions and the *p10* coding region close to TSS (Figure 4D,E). The mapping pattern is provided in Supplementary Figure S2. Before ChIP-qPCR, the sequence-amplifying primers within or close to the *polh* and *p10* promoter regions were designed (Figure 4D,E) for PCR detection, so as to determine the primer specificity to ensure their accuracy (Supplementary Figure S3). Our results demonstrated that actin was highly enriched by 114.63 and 274.54 folds in the *polh* and *p10* promoter regions (F3) compared with the IgG control, respectively. Interestingly, the enrichment degree gradually increased from F1 to F3, and greater enrichment was observed sites closer to the TSS. The actin-binding specificity to the polh promoter (F3) was the highest among the four regions. It appeared that actin tended to be predominantly enriched in the promoter region of polh, but actin also occurred in the *p10* gene body close to TSS (F4). Since actin lacks the intrinsic DNA-binding ability, the existing data suggest that actin indirectly binds to *polh* and *p10* promoter regions by interacting with viral polymerase. Combined with the interactions between actin and viral polymerase, we concluded that actin was a component of *polh* and *p10* TIC.

### 3.5. Actin Interacted with Transcription Regulators and Might Tether TIC Formation

Accumulating evidence suggests that transcription initiation is very complex and requires multiple factors to participate in the process. Given that actin dynamics is essential for viral polymerase recruitment, we wondered whether actin may function as a scaffold to organize TIC. To test this hypothesis, we sought to identify the relationships between actin and three key regulators IE1, PK1, and VLF-1. IE1 is highly conserved in all baculoviruses and considered as the major transcriptional trans-activator necessary for viral gene expression [41]. PK1, a baculovirus-encoded serine/threonine kinase, is a component of the very late gene TIC [42]. The polh mRNA and protein expression levels are inhibited by the knockdown of PK1 [43]. In addition, late and very late promoters differ primarily in the presence or absence of a burst sequence, a sequence in the downstream transcriptional start site (namely, 90% A + T) [24,25,26,27]. A viral gene required for the burst of very late gene transcription is called very late expression factor 1 (*vlf-1*), which stimulates expression from *polh* and *p10* promoters [44]. VLF-1 specifically transactivates *polh* and *p10* by binding to the burst sequence [36].

**Figure 5:**
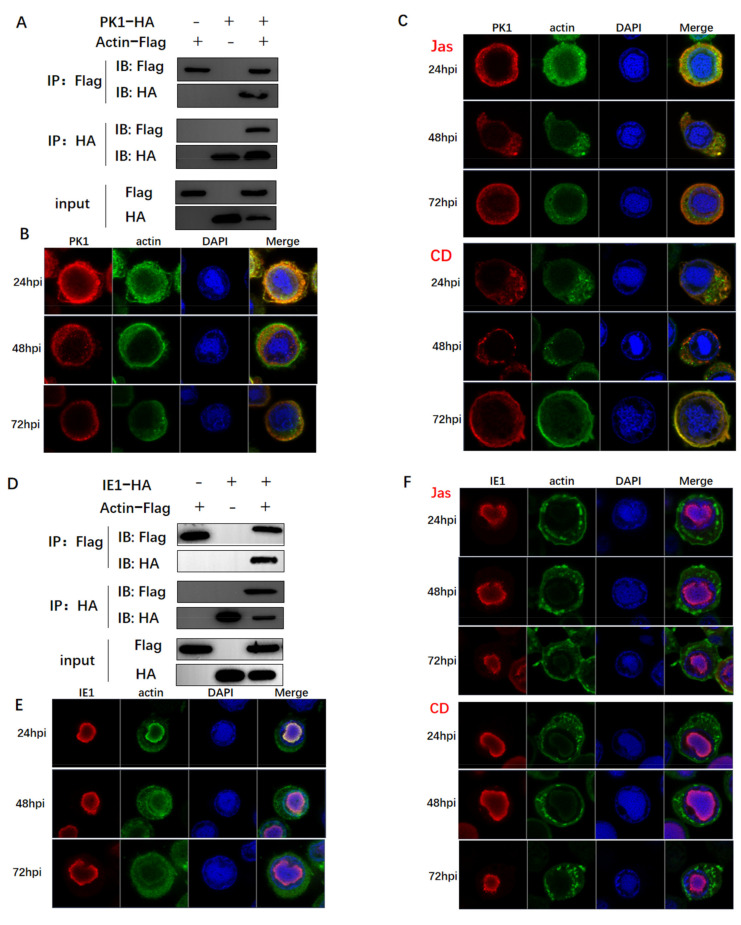

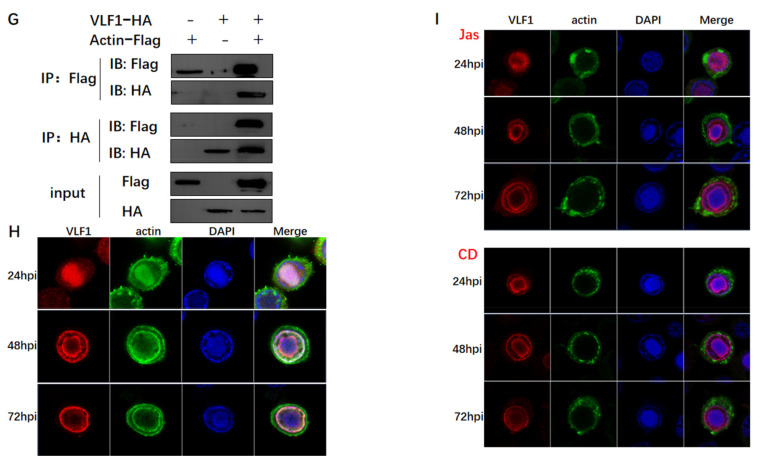
The analysis of the relationship between actin and transcriptional regulators. (**A**,**D**,**G**) Co-IP assays used to confirm interactions of actin with PK1 and IE1. The method was the same as in Figure 3. (**B**,**E**,**H**) Co-localization analysis of actin with PK1 and IE1 by confocal microscopy. The method was the same as in Figure 3. (**C**,**F**,**I**) As in (**B**,**E**,**H**), at 15 h p.i., cells were treated with Jas or CD and then subjected to confocal microscopy.

As found from Co-IP assays, actin interacted and was colocalized with PK1, IE1, and VLF-1 (Figure 5A,B,D,E,G,H). In addition to the attenuated nuclear distribution of viral polymerase, both CD and Jas treatments also caused dramatic redistribution of PK1 into the highly concentrated nuclear member foci (Figure 5C), thereby raising the possibility that interaction with PK1 might promote PK1 nuclear translocation. As shown in Figure 4C, CD and Jas treatments decreased the nuclear accumulation of PK1 at 24 h p.i., accompanied by the phenomenon that actin was obviously clustered outside the nuclear membrane. The localization of IE1 and VLF1 was not affected by CD or Jas treatment, despite the apparently clustered actin distribution in the cytoplasm (Figure 5E,F). It might be because IE1 and VLF-1 translocated to nucleus with different powers.

### 3.6. Overexpression of Actin Stimulated the Transcription of polh and p10 Promoters

**Figure 6:**
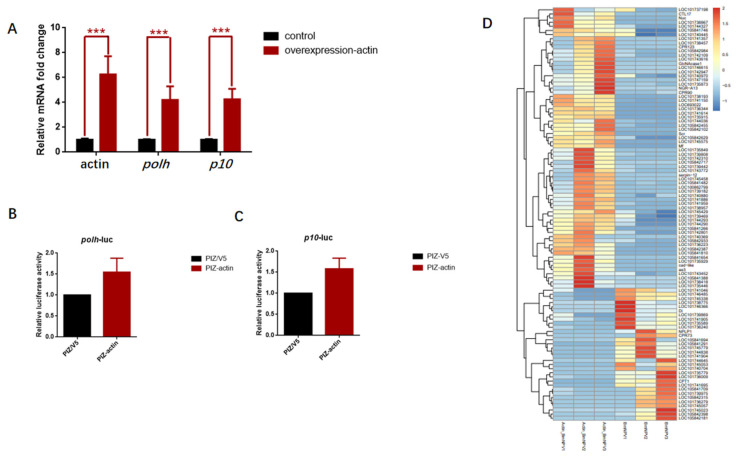

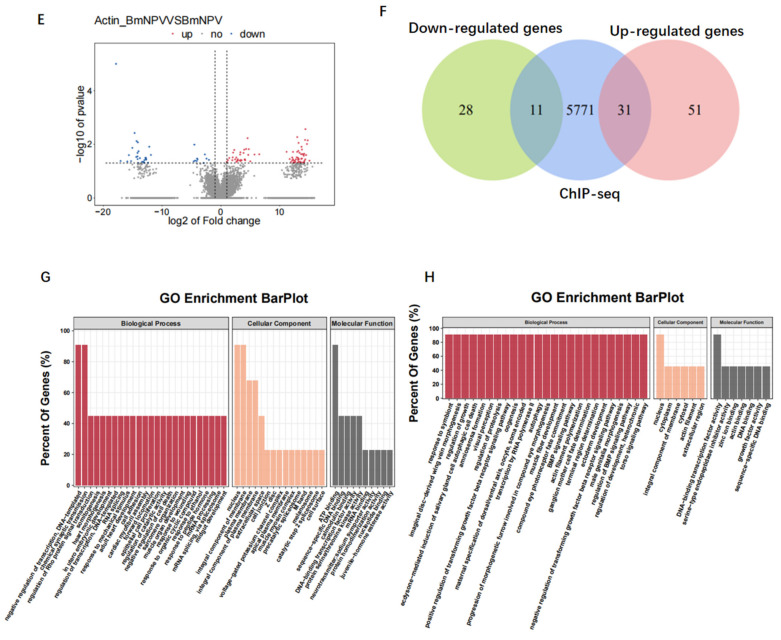
Overexpression of actin stimulates *polh* and *p10* transcription. (**A**) Quantitative real-time PCR analysis of actin, *polh*, and *p10* in BmN cells overexpressing actin or empty vector (Ctrl) (*n* = 3, ***, *p* < 0.001). (**B**,**C**) The relative luciferase expression activity under the control of *polh* and *p10*. (**D**) Heatmap showing the fold change for differentially expressed host genes in cells overexpressing actin and control cells expressing empty vector. (**E**) Scatter plot of global mRNA expression in cells overexpressing actin and control by polyA(+) RNA-seq. Number of up- and downregulated mRNAs is shown. (**F**) Venn diagram representing overlap between differentially expressed genes (up and down) and the enrichment host genes of actin from ChIP-seq. (**G**,**H**) The functional annotations of the overlapped genes in (**E**).

To gain further insight into how actin regulated gene transcription, the transient expression vector pIZ-actin-Flag was transfected to further verify the regulatory function of actin. The pIZ-actin-Flag recombinant plasmid and pIZ empty vector were transfected into the cultured BmN cells. Later, cells were further infected with BmNPV at 12 h post-transfection, when actin was abundantly expressed. Based on the RT-PCR results, the *polh* and *p10* transcriptional levels were significantly upregulated at 48 h p.i. (Figure 6A). To demonstrate the general applicability, Luc activity was employed to detect the effect of actin overexpression on promoter activity. Therefore, Luc was expressed in the *polh* and *p10* promoters on the pFast-HTB and pFast-Dual vectors commonly used in BEVS. Afterward, cells transfected with pIZ-actin-Flag and pIZ empty vector were further infected with recombinant BmNPV containing the genes Luc, vBm-*polh*-Luc, and vBm-*p10*-Luc. Cell lysates were collected at 48 h p.i., and chemiluminescence was detected. According to our results, the Luc activity increased by 54.50% in vBm-*polh*-Luc and 58.09% in vBm-*p10*-Luc compared with the control (Figure 6B,C).

These results suggested that actin upregulated the *polh* promoter-driven transcription in BmN cells. The addition of actin stimulated Luc expression in BEVS, which further confirmed the idea that actin promoted the transcription of *polh* and *p10* promoters. Based on the above results, we concluded that actin functioned as a transcriptional activator that regulated viral late gene transcription.

### 3.7. Baculovirus Might Hijack Actin of the Host Transcription Apparatus

Expression of host genes is shut off in the late stage of baculovirus infection. To determine whether overexpression of actin induced transcriptional changes of host genes, we conducted RNA sequencing (RNA-seq) analysis (Figure 6D,E). The RNA-seq results indicated that actin had both positive and negative effects on the expression of host genes, including 82 upregulated and 39 downregulated ones. Moreover, we identified 5813 host genes with actin-binding sites from ChIP-seq, and 4.69% of them were in the promoter regions (Supplementary Figure S4). Combined with RNA-seq and ChIP-seq data, both upregulated and downregulated genes considerably overlapped with actin-binding genes (Figure 6F). Further, GO and KEGG pathway enrichment analyses demonstrated that differentially expressed genes (DEGs) were mainly concentrated in biological processes such as negative regulation of transcription, DNA-templated synthesis, and transcription by RNA polymerase II. Additionally, analysis of the upregulated and downregulated genes revealed strong enrichments for DNA-binding transcription factor activity, actin binding, DNA binding, and sequence−specific DNA-binding-related molecular function (Figure 6G,H). These results suggested that baculovirus might hijack actin working for host gene transcription to assist in its gene expression.

## 4. Discussion

**Figure 7:**
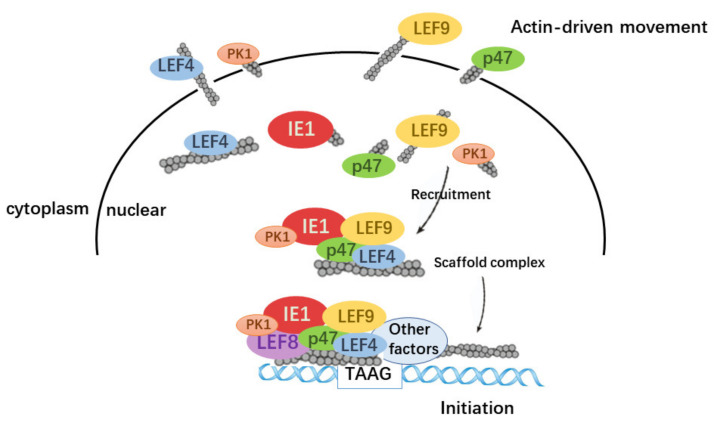
Summary model for the regulatory roles of actin play in baculovirus polymerase transcription. A model of how actin dynamics assists in the hyperexpression in the baculovirus expression vector system.

In this report, we discovered that actin played an important role in the hyperexpression of two very late genes, *polh* and *p10*. Actin has no inherent DNA-binding capability, but it is enriched in the *polh* and *p10* promoters via promoter-binding partners. The interactions of actin with components of viral polymerase TIC enable actin to serve as a scaffolding molecule to facilitate TIC formation (Figure 7). Recent results have demonstrated that actin executes similar functions during the serum-induced transcriptional program, where actin contributes to Pol II transcription by scaffolding the larger, more active, and more long-lasting Pol II clusters upon serum stimulation [45].

Firstly, we detected the direct correlations between the transcripts of *polh* and *p10* and actin dynamics. According to our results, the disruption of actin dynamics by CD and Jas treatments delayed the transcription of *polh* and *p10*, indicating that the baculovirus-encoded polymerase was actin-sensitive (Figure 2). Co-IP assays and confocal microscopy clearly indicated that actin interacted and was colocalized with three subunits of viral RNA polymerase, and nuclear import of these three subunits was prohibited with the disruption of actin dynamics (Figure 3). Accumulating evidence has suggested that nuclear G- and F-actin are in a dynamic state, which provides power for the import and export of transcription factors [46]. Therefore, we concluded that actin dynamics may provide forces for viral polymerase movement, which thus affected viral polymerase recruitment.

Secondly, the high enrichment of actin within the promoter sequences of *polh* and *p10* further suggested actin as a component of viral polymerase TIC, as revealed by ChIP-seq and ChIP-qPCR results (Figure 4E,F). It is known that the *polh* and *p10* promoters possess the conserved sequences essential for their expression. The viral very late promoters are transcribed by viral RNA polymerase and contain a TAAG sequence motif. TSS is always initiated at the second A in the TAAG motif. Moreover, three of the peak concentrations all showed an AAG motif (Figure 4D), clearly implying that actin participated in viral RNA polymerase transcription. The regulation of transcription initiation is a very complex process involving a wide variety of proteins with large molecular weights and highly dynamic structural forms. We performed further studies to examine the relationships between actin and transcriptional regulators PK1, IE1, and VLF-1. Surprisingly, actin interacted and was colocalized with the three essential transcription regulators (Figure 5A,B,D,E,G,H). In addition, the destruction of actin dynamics drove the relocalization of PK1, with decreased nuclear accumulation of PK1 (Figure 5C). It was reported that PK1 was a component of the very late gene TIC, which interacted with the very late promoters containing 5′ UTR to regulate the hyperexpression of very late genes [42]. Moreover, PK1 phosphorylates LEF8 [47]. The C-terminal region (CTR) of RNA polymerase II is phosphorylated, thus facilitating the conversion to transcriptional elongation [48]. As for a subunit of viral polymerase, the phosphorylation of LEF8 may exert the same effect on the transcriptional elongation of viral polymerase. To some extent, actin participated in transcriptional regulation by controlling the accessibility of PK1. The association and colocalization apparently existed between actin and IE1 as well as VLF1, although the distribution of IE1 and VLF-1 was not affected by actin dynamics. However, IE1 and VLF-1 were components of TIC, and VLF-1 stimulated *polh* and *p10* expression by binding to their promoters. Through interactions with TIC components, actin might exert its function as a scaffold protein for the assembly of the transcription complex. Moreover, other viral proteins that affected *polh* and *p10* expression and host proteins not confirmed yet might be assembled or gathered together by the actin scaffold to participate in gene regulation.

Finally, we surprisingly found that overexpression of actin dramatically increased the transcript amounts of *polh* and *p10* (Figure 6A). The addition of actin stimulated Luc expression in BEVS (Figure 6B,C). In this regard, results from luciferase reporter assay implied that actin functioned as a transcriptional activator for the *polh* and *p10* promoters. Thereafter, this study conducted an association analysis on RNA-seq and ChIP-seq data of actin-overexpression-induced host genes. GO and KEGG analyses indicated that baculovirus might hijack actin of the host transcription apparatus to facilitate its own transcription.

In summary, our present findings supported the notion that actin positively regulated the expression of *polh* and *p10*. The concentrations of transcription factors and co-factors close to the transcription initiation sites represent a sensitive limiting component that determines the number of transcripts produced. Overexpression of actin may enhance viral polymerase movement and then accelerate the recruitment of viral polymerase in the upstream transcription site, which facilitates TIC formation.

## Data Availability

The ChIP-seq data and RNA-seq data generated in this study have been submitted to the NCBI BioProject database (https://www.ncbi.nlm.nih.gov/bioproject/, accessed on 1 December 2021) under accession number PRJNA735791 and PRJNA735599.

## Supplementary Materials

The following are available online at https://www.mdpi.com/article/10.3390/v14010153/s1, Figure S1: The timing of nuclear recruitment and polymerization of actin, Figure S2: The *polh* and *p10* mapping pattern of ChIP-seq reads, Figure S3: The test of primers used in ChIP-qPCR, Figure S4: ChIP-seq on host gene, Table S1: Sequences of the DNA primers for biotined promoters, Table S2: Sequences of the DNA primers for RT-qPCR, Table S3: Primers used in Co-IP assays, Table S4: Sequences of the DNA primers for ChIP-qRCR, Table S5: Sequences of the DNA primers for amplification, Table S6: List of potential polh and p10 interacting proteins of BmNPV identified by LC-MS/MS, Table S7: List of potential polh and p10 interacting proteins of host identified by LC-MS/MS, Table S8: List of actin binding sites were identified in viral genome from ChIP-seq.
