# Supplementary material for: Actin Contributes to the Hyperexpression of Baculovirus Polyhedrin (polh) and p10 as a Component of Transcription Initiation Complex (TIC)
Source: Viruses. 2022 Jan 14;14(1):153. doi: 10.3390/v14010153 (PMC8779803; doi:10.3390/v14010153)
Supplement: Supplementary file 1 [file viruses-14-00153-s001.zip › viruses-1521103-supplementary.pdf]

**Supplementary Results**

Table S1. Sequences of the DNA primers for biotined promoters

| Name | Sequence（5’-3’） |
|---|---|
| polh | Forward: GCTGGCAACTGCAAGGGCCTC<br>Reverse: GGTGTATGAATAATTCGGCAT |
| P10 | Forward: GAAATTATGCATTTGAGGATG<br>Reverse: GTGTCAAAACGTTAGGCTTTG |

Table S2. Sequences of the DNA primers for RT-qPCR

| Name | Sequence（5’-3’） |
|---|---|
| polh | Forward: AGGTGCTACAAGTTCCTCGC<br>Reverse: TCGTTGTTCATGCCCACGTA |
| P10 | Forward: AACGGGCTGGAAGAATCGTT<br>Reverse: GAGCAGTGTCACCGGTCAAT |
| 39K | Forward: TGCAGGACGAACGCAAAATG<br>Reverse: AAAATCTGCCAATTCGGGCG |
| 28S rRNA | Forward: CGACGTTGCTTTTTGATCCT<br>Reverse: GCAACGACAAGCCATCAGTA |
| Actin | Forward: GCCTCCCTCTCTACCTTCCA<br>Reverse: CAAGCCTGAATTGAGCAGCC |

Table S3. Primers used in Co-IP assays

| Name | Sequence（5’-3’）Underlined sequences indicate the enzyme digestion site for each primer. |
|---|---|
| P47-HA | Forward: gggATCCATGTTTGTCACCCG<br>Reverse:<br>gAATTCTCAAGCGTAATCTGGAACATCGTATGGGTAGTATAACGACTGGTCAAAATTG |
| LEF4-HA | Forward: gggATCCATGGACCACGGCAATTTTATG<br>Reverse:<br>gAATTCTTAAGCGTAATCTGGAACATCGTATGGGTAATTTGGCACGATTCGGTCGCGAC |
| LEF8-HA | Forward: gggATCCATGACGGACGTAGTTCAAGATTTCAACGAG<br>Reverse:<br>gAATTCTCAAGCGTAATCTGGAACATCGTATGGGTAATTTTTCATTATCGTATTGTTGC |
| LEF9-HA | Forward: gggATCCATGTTTTCTTTTTTGGATAAAACTCCTACTGAG<br>Reverse:<br>gAATTCTCAAGCGTAATCTGGAACATCGTATGGGTATTCAATAAACATGTCGAGC |
| PK1-HA | Forward: gggATCCATGGCCACCACCACCGCCACAAATGC |

| | |
|---|---|
| | Reverse: gAATTCTCAAGCGTAATCTGGAACATCGTATGGGTACGACAAAAACTCATG |
| IE1-HA | Forward: gggATCC ATGACGCAAATTAATTTTAACGCGTCGTACACCAGTGCTCCG<br>Reverse: gAATTCTTAAGCGTAATCTGGAACATCGTATGGGTAATTAAATTCAATTTTTTTAT |
| VLF1-HA | Forward:gggATCCATGAACGGTTTTAATGTTCGCAACG<br>Reverse: gAATTCCTAAGCGTAATCTGGAACATCGTATGGGTATTCGTTGCGATAGTACAACAACG |
| Actin-Flag | Forward: gggATCCATGTGCGACGAAGAAGTTGCC<br>Reverse: gAATTCTTACTTATCGTCGTCATCCTTGTAATCGAAGCACTTCCTGTGTAC |

Restriction enzyme sites are underlined. *BamH*I, GGATCC; *EcoR*I, GAATTC

## Table S4. Sequences of the DNA primers for ChIP-qRCR

| Name | Sequence（5’-3’） |
|---|---|
| Polh F1 | Forward: AGGCTTTGGAGTTTACTACAGA<br>Reverse: TAACATACAACGCTTTTGATTT |
| Polh F2 | Forward: GTTTTGAGCGAAGACGGC<br>Reverse: ATTATTCACGATTGCCGCT |
| Polh F3 | Forward: TTACAACAGGGGGGACTATG<br>Reverse:ACCTTTGTGTTAGTTTCCGC |
| Polh F4 | Forward:TGACCGGTGACACTGCTC<br>Reverse:GATTTATTTACTTGGAACTGCG |
| P10 F1 | Forward:TTCAGAGAAACCAGTCGCC<br>Reverse:GACGCCCAAGCGACAGTA |
| P10 F2 | Forward:GTGGAGTCCTCTCATTAGCG<br>Reverse:CGCCATTGTAGAATACCGTG |
| P10 F3 | Forward:CCATCTCGCAAATAAATAAG<br>Reverse:CATAAGAGTCCTTCTGTCCC |
| P10 F4 | Forward:CGTGATGGTCGGTATGGG<br>Reverse:CCCAGTTAGTGACGATTCCG |

## Table S5. Sequences of the DNA primers for amplification

| **Applications** | Name | Sequence（5’-3’） |
|---|---|---|
| Amplification of luc for pFastBac HTB plasmid | pFast-HTB-luc | Forward:GGATCCGGCAATCCGGTACTGTTGGTAAAGC<br>Reverse:GAATTCACGGCGATCTTGCCGCCCTTCTTGGCCTTAAT |

| | | |
|---|---|---|
| Amplification of luc for pFastBac Dual plasmid | pFast-Dual-luc | Forward:CTCGAGGGCAATCCGGTACTGTTGGTAAAGC<br>Reverse:GGTACCACGGCGATCTTGCCGCCCTTCTTGGCCTTAAT |
| Amplification of actin for PIZ-v5 plasmid | PIZ-actin | Forward: GGATCCATGTGCGACGAAGAAGTTGCC<br>Reverse:GAATTCTTACTTATCGTCGTCATCCTTGTAATCGAAGCACTTCCTGTGTAC |

Restriction enzyme sites are underlined. *BamH*I, GGATCC; *EcoR*I, GAATTC; *Xho*I, CTCGAG; *Kpn*I, GGTACC

Table S6. List of potential polh and p10 interacting proteins of BmNPV identified by LC-MS/MS.

| Accession | Description | Coverage | Unique Peptides | Peptides |
|---|---|---|---|---|
| O92432 | LEF-3 OS=Bombyx mori nuclear polyhedrosis virus OX=271108 GN=lef-3 PE=4 SV=1 - [O92432_NPVBM] | 43.38 | 1 | 19 |
| O92407 | p47 OS=Bombyx mori nuclear polyhedrosis virus OX=271108 GN=p47 PE=4 SV=1 - [O92407_NPVBM] | 39.85 | 18 | 18 |
| I7A7I0 | p6.9 OS=Bombyx mandarina nucleopolyhedrovirus S2 OX=1208064 GN=p6.9 PE=4 SV=1 - [I7A7I0_NPVBM] | 27.69 | 1 | 2 |
| I7B1P8 | BV/ODV-NC50 OS=Bombyx mandarina nucleopolyhedrovirus S2 OX=1208064 GN=bv PE=4 SV=1 - [I7B1P8_NPVBM] | 22.90 | 13 | 14 |
| I7ASQ0 | Alkaline nuclease OS=Bombyx mandarina nucleopolyhedrovirus S2 OX=1208064 GN=Bomanpvs2gp115 PE=4 SV=1 - [I7ASQ0_NPVBM] | 22.43 | 1 | 13 |
| I6ZWQ6 | 39K protein OS=Bombyx mandarina nucleopolyhedrovirus S2 OX=1208064 GN=Bomanpvs2gp028 PE=4 SV=1 - [I6ZWQ6_NPVBM] | 20.94 | 6 | 6 |
| K4GHJ0 | VLF-1 OS=Bombyx mori nuclear polyhedrosis virus OX=271108 GN=Bmnpvindiagp065 PE=4 SV=1 - [K4GHJ0_NPVBM] | 20.16 | 11 | 11 |
| I7B5F3 | Ubiquitin OS=Bombyx mandarina nucleopolyhedrovirus S2 OX=1208064 GN=Bomanpvs2gp027 PE=4 SV=1 - [I7B5F3_NPVBM] | 19.48 | 2 | 2 |

| | | | | |
|---|---|---|---|---|
| O92415 | LEF-8 OS=Bombyx mori nuclear polyhedrosis virus OX=271108 GN=lef-8 PE=4 SV=1 - [O92415_NPVBM] | 5.02 | 6 | 6 |
| O92427 | LEF-9 OS=Bombyx mori nuclear polyhedrosis virus OX=271108 GN=lef-9 PE=4 SV=1 - [O92427_NPVBM] | 3.67 | 3 | 3 |

Table S7. List of potential polh and p10 interacting proteins of host identified by LC-MS/MS.

| Accession | Description | Coverage | Unique Peptides | Peptides |
|---|---|---|---|---|
| S5M6C1 | Actin-4 OS=Bombyx mori OX=7091 PE=2 SV=1 - [S5M6C1_BOMMO] | 42.05 | 18 | 18 |
| H9J7S2 | Ribosome biogenesis regulatory protein OS=Bombyx mori OX=7091 GN=101743869 PE=3 SV=1 - [H9J7S2_BOMMO] | 41.76 | 15 | 14 |
| H9JYF8 | H15 domain-containing protein OS=Bombyx mori OX=7091 GN=101740214 PE=3 SV=1 - [H9JYF8_BOMMO] | 40.71 | 10 | 10 |
| H9JBE0 | Uncharacterized protein OS=Bombyx mori OX=7091 PE=3 SV=1 - [H9JBE0_BOMMO] | 34.86 | 13 | 13 |
| O76149 | Tubulin beta chain OS=Bombyx mori OX=7091 GN=bmtub2 PE=2 SV=1 - [Q8T8B2_BOMMO] | 32.44 | 6 | 13 |
| H9JB82 | Protein kinase domain-containing protein OS=Bombyx mori OX=7091 PE=3 SV=1 - [H9JB82_BOMMO] | 30.30 | 13 | 13 |
| H9JX90 | KH domain-containing protein OS=Bombyx mori OX=7091 PE=4 SV=1 - [H9JX90_BOMMO] | 29.97 | 13 | 13 |
| H9JCL8 | Uncharacterized protein OS=Bombyx mori OX=7091 PE=4 SV=1 - [H9JCL8_BOMMO] | 26.75 | 12 | 12 |
| H9J271 | Elongation factor 1-alpha OS=Bombyx mori OX=7091 GN=EF1a PE=2 SV=1 - [H9J271_BOMMO] | 25.92 | 12 | 13 |
| B9VS67 | HnRNPA/B-like 28 OS=Bombyx mori OX=7091 GN=hrp28 PE=2 SV=1 - [B9VS67_BOMMO] | 23.83 | 2 | 5 |
| H9J789 | KRR1 small subunit processome component OS=Bombyx mori OX=7091 GN=101743195 PE=3 SV=1 - [H9J789_BOMMO] | 23.49 | 7 | 7 |
| H9JVK4 | Uncharacterized protein OS=Bombyx mori OX=7091 PE=3 SV=1 - [H9JVK4_BOMMO] | 23.00 | 12 | 12 |
| H9JSJ2 | Uncharacterized protein OS=Bombyx mori OX=7091 PE=4 SV=1 - [H9JSJ2_BOMMO] | 22.65 | 12 | 12 |

| | | | | |
|---|---|---|---|---|
| H9JSP8 | Uncharacterized protein OS=Bombyx mori OX=7091 GN=101740273 PE=4 SV=1 - [H9JSP8_BOMMO] | 20.45 | 10 | 10 |
| H9J7G0 | Ribosome biogenesis protein WDR12 homolog OS=Bombyx mori OX=7091 GN=101736588 PE=3 SV=1 - [H9J7G0_BOMMO] | 19.76 | 8 | 8 |
| H9IYV4 | Uncharacterized protein OS=Bombyx mori OX=7091 GN=101741225 PE=4 SV=1 - [H9IYV4_BOMMO] | 19.51 | 9 | 9 |
| H9JSN0 | Uncharacterized protein OS=Bombyx mori OX=7091 PE=3 SV=1 - [H9JSN0_BOMMO] | 17.96 | 6 | 6 |
| H9JR08 | Uncharacterized protein OS=Bombyx mori OX=7091 PE=3 SV=1 - [H9JR08_BOMMO] | 16.24 | 8 | 11 |
| H9JJ94 | WD_REPEATS_REGION domain-containing protein OS=Bombyx mori OX=7091 PE=4 SV=1 - [H9JJ94_BOMMO] | 16.02 | 7 | 7 |
| H9JR28 | RRM domain-containing protein OS=Bombyx mori OX=7091 PE=4 SV=1 - [H9JR28_BOMMO] | 14.82 | 9 | 9 |
| H9JMI6 | Nop domain-containing protein OS=Bombyx mori OX=7091 PE=4 SV=1 - [H9JMI6_BOMMO] | 14.52 | 7 | 7 |
| H9JTB0 | Uncharacterized protein OS=Bombyx mori OX=7091 GN=101741115 PE=4 SV=1 - [H9JTB0_BOMMO] | 14.35 | 7 | 7 |
| Q5UAT7 | Ribosomal protein L4 OS=Bombyx mori OX=7091 GN=RpL4 PE=2 SV=1 - [Q5UAT7_BOMMO] | 13.08 | 6 | 7 |
| H9J7A3 | Uncharacterized protein OS=Bombyx mori OX=7091 PE=4 SV=1 - [H9J7A3_BOMMO] | 12.64 | 5 | 5 |
| H9JR70 | Uncharacterized protein OS=Bombyx mori OX=7091 PE=4 SV=1 - [H9JR70_BOMMO] | 12.54 | 4 | 4 |
| H9J5J4 | Protein CLP1 homolog OS=Bombyx mori OX=7091 PE=3 SV=1 - [H9J5J4_BOMMO] | 12.31 | 6 | 6 |
| H9JXB6 | Uncharacterized protein OS=Bombyx mori OX=7091 PE=3 SV=1 - [H9JXB6_BOMMO] | 11.20 | 6 | 6 |
| H9JDE4 | Uncharacterized protein OS=Bombyx mori OX=7091 PE=4 SV=1 - [H9JDE4_BOMMO] | 10.33 | 4 | 4 |
| H9JK50 | Uncharacterized protein OS=Bombyx mori OX=7091 GN=101738695 PE=4 SV=1 - [H9JK50_BOMMO] | 10.31 | 5 | 5 |
| H9IS99 | Uncharacterized protein OS=Bombyx mori OX=7091 PE=4 SV=1 - [H9IS99_BOMMO] | 10.06 | 2 | 2 |
| H9JA60 | Uncharacterized protein OS=Bombyx mori OX=7091 PE=4 SV=1 - [H9JA60_BOMMO] | 9.14 | 4 | 4 |

| | | | | |
|---|---|---|---|---|
| H9J2U6 | PUA domain-containing protein OS=Bombyx mori OX=7091 GN=101744752 PE=4 SV=1 - [H9J2U6_BOMMO] | 8.82 | 5 | 5 |
| H9J150 | G-patch domain-containing protein OS=Bombyx mori OX=7091 GN=101739259 PE=4 SV=1 - [H9J150_BOMMO] | 7.27 | 3 | 3 |
| H9JT95 | Uncharacterized protein OS=Bombyx mori OX=7091 PE=4 SV=1 - [H9JT95_BOMMO] | 7.17 | 2 | 2 |
| H9JJI4 | RRM domain-containing protein OS=Bombyx mori OX=7091 GN=101746268 PE=4 SV=1 - [H9JJI4_BOMMO] | 6.41 | 5 | 5 |
| H9JR08 | Uncharacterized protein OS=Bombyx mori OX=7091 PE=3 SV=1 - [H9JR08_BOMMO] | 8.44 | 6 | 6 |
| H9JKX7 | Uncharacterized protein OS=Bombyx mori OX=7091 PE=4 SV=1 - [ H9JKX7_BOMMO] | 8.96 | 4 | 4 |

Table S8. List of actin binding sites were identified in viral genome from ChIP-seq

| Gene | Gene Inside |
|---|---|
| Bmnpvgp003 | Bmnpvgp003:NC_001962.1:2394-3222:+:-646 |
| Bmnpvgp004 | Bmnpvgp004:NC_001962.1:3247-4270:-:395 |
| Bmnpvgp006 | Bmnpvgp006:NC_001962.1:5479-6292:-:164 |
| Bmnpvgp007 | Bmnpvgp007:NC_001962.1:6406-7927:+:494 |
| Bmnpvgp012 | Bmnpvgp012:NC_001962.1:10458-10791:+:864 |
| Bmnpvgp014 | Bmnpvgp014:NC_001962.1:12334-13483:+:621 |
| Bmnpvgp017 | Bmnpvgp017:NC_001962.1:16185-17139:-:259 |
| Bmnpvgp020 | Bmnpvgp020:NC_001962.1:18488-19010:+:-90 |
| Bmnpvgp021 | Bmnpvgp021:NC_001962.1:19128-19344:-:-610 |
| Bmnpvgp023 | Bmnpvgp023:NC_001962.1:20851-21805:-:466 |
| Bmnpvgp026 | Bmnpvgp026:NC_001962.1:23406-23955:+:214 |
| Bmnpvgp027 | Bmnpvgp027:NC_001962.1:24366-25014:-:217 |
| Bmnpvgp029 | Bmnpvgp029:NC_001962.1:25320-26154:-:498 |
| Bmnpvgp030 | Bmnpvgp030:NC_001962.1:26147-26486:-:-199 |
| Bmnpvgp033 | Bmnpvgp033:NC_001962.1:28265-29465:-:449 |
| Bmnpvgp035 | Bmnpvgp035:NC_001962.1:30151-31672:+:328 |
| Bmnpvgp036 | Bmnpvgp036:NC_001962.1:31685-31922:+:-52 |
| Bmnpvgp039 | Bmnpvgp039:NC_001962.1:32865-34974:+:1057 |
| Bmnpvgp040 | Bmnpvgp040:NC_001962.1:35072-35342:-:-894 |
| Bmnpvgp041 | Bmnpvgp041:NC_001962.1:35593-38227:-:693 |

| | |
|---|---|
| Bmnpvgp046 | Bmnpvgp046:NC_001962.1:40299-41397:+:68 |
| Bmnpvgp049 | Bmnpvgp049:NC_001962.1:42220-42706:+:503 |
| Bmnpvgp052 | Bmnpvgp052:NC_001962.1:43653-44298:-:277 |
| Bmnpvgp054 | Bmnpvgp054:NC_001962.1:45934-46402:+:-747 |
| Bmnpvgp055 | Bmnpvgp055:NC_001962.1:46478-47363:-:398 |
| Bmnpvgp056 | Bmnpvgp056:NC_001962.1:47492-50453:-:1117 |
| Bmnpvgp058 | Bmnpvgp058:NC_001962.1:52881-54039:-:1096 |
| Bmnpvgp060 | Bmnpvgp060:NC_001962.1:54439-55228:+:-45 |
| Bmnpvgp062 | Bmnpvgp062:NC_001962.1:56184-56367:+:-315 |
| Bmnpvgp067 | Bmnpvgp067:NC_001962.1:58189-59329:-:261 |
| Bmnpvgp072 | Bmnpvgp072:NC_001962.1:61737-62283:-:-566 |
| Bmnpvgp076 | Bmnpvgp076:NC_001962.1:67519-68572:-:97 |
| Bmnpvgp081 | Bmnpvgp081:NC_001962.1:71756-72443:+:-164 |
| Bmnpvgp081 | Bmnpvgp081:NC_001962.1:71756-72443:+:1423 |
| Bmnpvgp083 | Bmnpvgp083:NC_001962.1:76131-76680:+:-1773 |
| Bmnpvgp083 | Bmnpvgp083:NC_001962.1:76131-76680:+:-267 |
| Bmnpvgp085 | Bmnpvgp085:NC_001962.1:77569-78526:+:718 |
| Bmnpvgp090 | Bmnpvgp090:NC_001962.1:81698-82070:-:67 |
| Bmnpvgp095 | Bmnpvgp095:NC_001962.1:87451-87769:-:-544 |
| Bmnpvgp099 | Bmnpvgp099:NC_001962.1:90088-91363:-:727 |
| Bmnpvgp100 | Bmnpvgp100:NC_001962.1:91384-91999:-:218 |
| Bmnpvgp103 | Bmnpvgp103:NC_001962.1:92531-94115:+:533 |
| Bmnpvgp107 | Bmnpvgp107:NC_001962.1:94758-95436:-:134 |
| Bmnpvgp109 | Bmnpvgp109:NC_001962.1:96375-97059:-:-802 |
| Bmnpvgp112 | Bmnpvgp112:NC_001962.1:99843-101436:-:557 |
| Bmnpvgp116 | Bmnpvgp116:NC_001962.1:103509-104172:+:-88 |
| Bmnpvgp117 | Bmnpvgp117:NC_001962.1:104199-105462:+:562 |
| Bmnpvgp121 | Bmnpvgp121:NC_001962.1:107701-108424:+:378 |
| Bmnpvgp123 | Bmnpvgp123:NC_001962.1:108795-110733:-:778 |
| Bmnpvgp124 | Bmnpvgp124:NC_001962.1:110963-112319:-:742 |
| Bmnpvgp125 | Bmnpvgp125:NC_001962.1:112595-113381:+:51 |
| Bmnpvgp126 | Bmnpvgp126:NC_001962.1:113395-114826:+:297 |
| Bmnpvgp128 | Bmnpvgp128:NC_001962.1:115153-116026:+:356 |
| Bmnpvgp130 | Bmnpvgp130:NC_001962.1:116322-116928:-:417 |

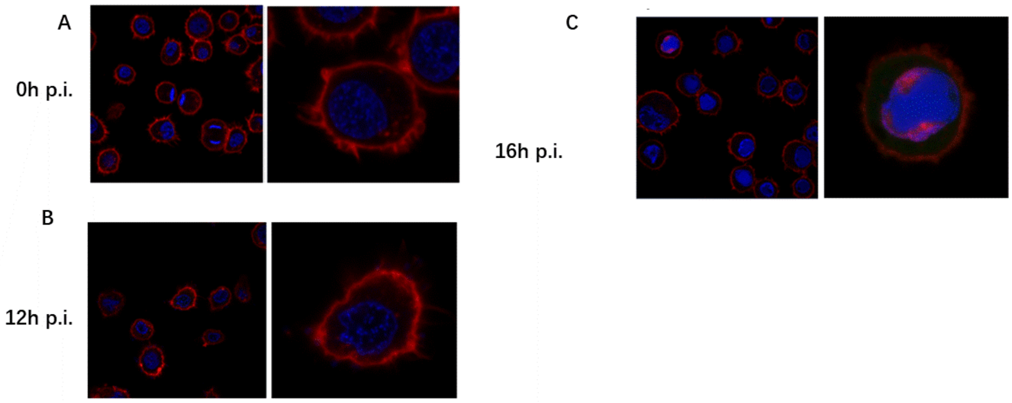


**Figure S1. The timing of nuclear recruitment and polymerization of actin**

(A) Staining of uninfected cells. F-actin was stained by YF594-Phalloidin (red) (Hangzhou Baimaihang Technology Co., Ltd.)

(B) Staining of Cells infected with BmNPV at 12h p.i..

(C) Staining of Cells infected with BmNPV at 16 p.i..

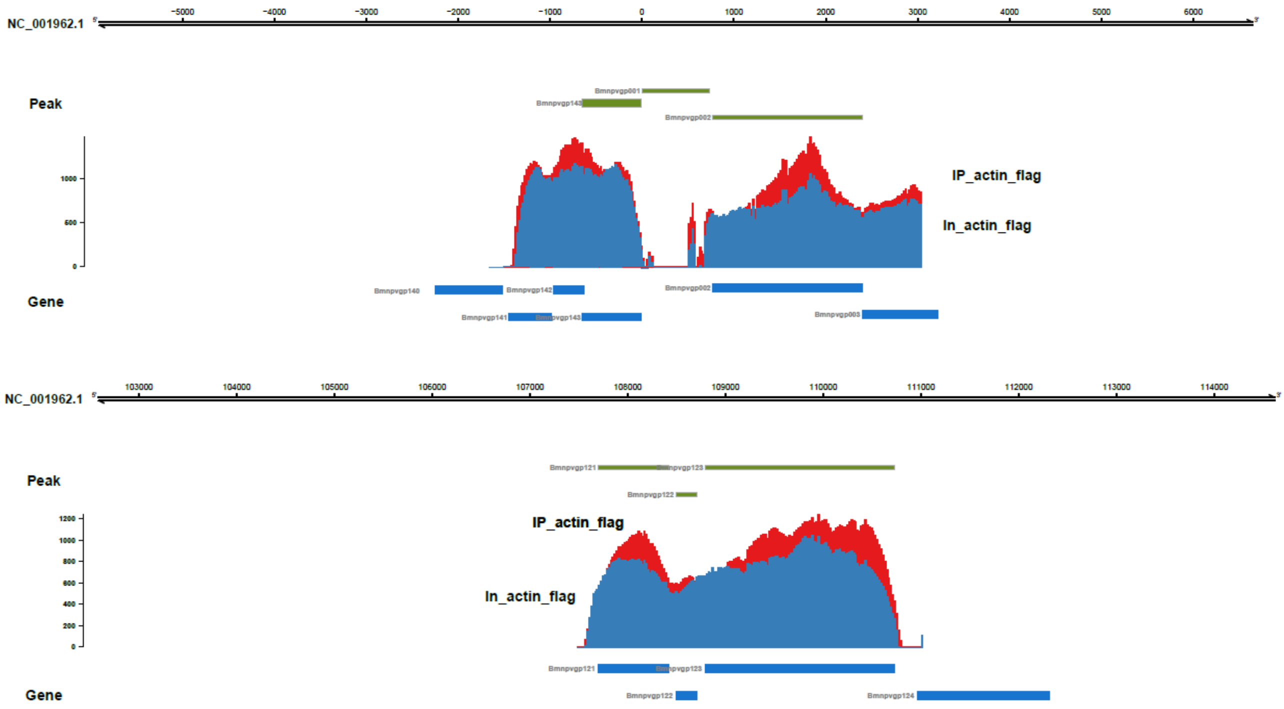


**Figure S2. The polh and p10 mapping pattern of ChIP-seq reads**

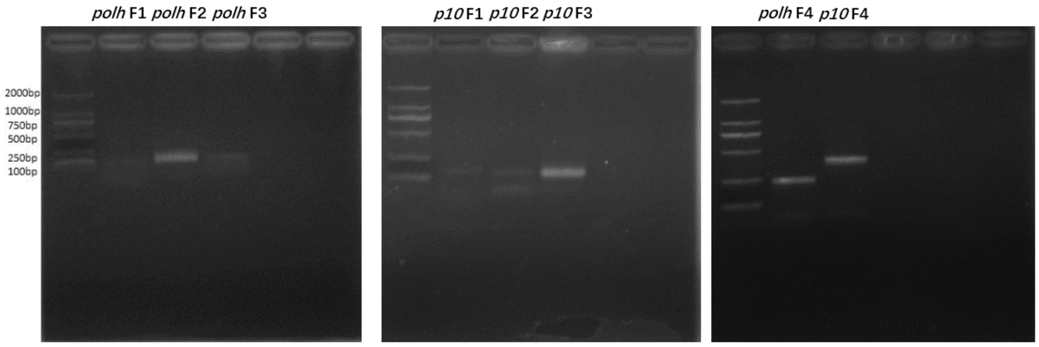


**Figure S3. The test of primers used in ChIP-qPCR**

The amplifying sequences within or close to the polh and p10 promoter region were designed for PCR detection.

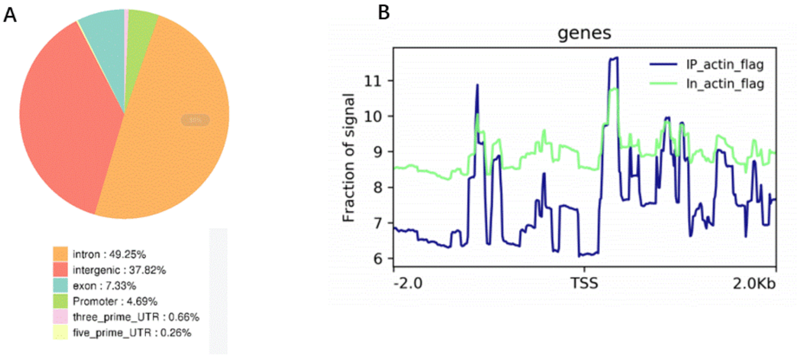


**Figure S4. ChIP-seq on host gene.**

(A) Statistics of distribution of actin binding sites in the host genome

(B) Distribution of actin binding sites in the host genic regions.
